# Localization and sub-cellular shuttling of HTLV-1 Tax with the microRNA machinery

**DOI:** 10.1186/1742-4690-11-S1-O73

**Published:** 2014-01-07

**Authors:** Rachel Van Duyne, Irene Guendel, Aarthi Narayanan, Kylene Kehn-Hall, Elizabeth Jaworski, Jessica Roman, William Coley, Zachary Klase, Anastas Popratiloff, Renaud Mahieux, Fatah Kashanchi

**Affiliations:** 1 School of Systems Biology, National Center for Biodefense & Infectious Diseases, George Mason University, Manassas, Virginia, USA; 2 Department of Microbiology, Immunology, & Tropical Medicine, The George Washington University Medical Center, Washington, D.C., USA; 3 Molecular Virology Section, Laboratory of Molecular Microbiology, National Institute of Allergy and Infectious Diseases, National Institutes of Health, Bethesda, Maryland, USA; 4 Department of Anatomy and Regenerative Biology, The George Washington University, Washington, D.C., USA; 5 Retroviral Oncogenesis Team, INSERM-U758 Virologie Humaine, Lyon, France

## 

The innate ability of the human cell to silence endogenous retroviruses through RNA sequences encoding microRNAs suggests that the cellular RNAi machinery is a major means by which the host mounts a defense response against retroviruses. Indeed, cellular miRNAs target and hybridize to specific sequences of both HTLV-1 and HIV-1 viral transcripts. However, the virus itself contains various mechanisms that assist in the evasion of viral inhibition through control of the cellular RNAi pathway. Retroviruses can hijack components of the RNAi pathway, in some cases to produce novel viral miRNAs that can either assist in active infection or promote a latent state. Here, we show that HTLV-1 Tax contributes to the dysregulation of the RNAi pathway by altering the expression of key components. A survey of uninfected and HTLV-1 infected cells revealed that Drosha is present at lower levels in all HTLV-1 infected cell lines and infected primary cells, while other components such as DGCR8 were not dramatically altered. We show co-localization of Tax and Drosha in the nucleus in vitro as well as co-immunoprecipitation in the presence of proteasome inhibitors, indicating that Tax interacts with Drosha and may target it to specific areas of the cell, namely, the proteasome. In the presence of Tax we observed a prevention of primary miRNA cleavage by Drosha. Finally, the changes in cellular miRNA expression in HTLV-1 infected cells can be mimicked by the add back of Drosha or the addition of antagomiRs against the cellular miRNAs which are downregulated by the virus.

